# Dark-Pi Imaging System Permits Open-Source Label-Free Microfluidic Monitoring of Platelet Aggregation by Cellular Light Scattering

**DOI:** 10.3390/s26144326

**Published:** 2026-07-08

**Authors:** Rüya Meltem Sarıyer Oglago, Alexander P. Bye, Sultan İlayda Dönmez Eryılmaz, Chris I. Jones, Alexander D. Edwards

**Affiliations:** 1 Department of Healthcare Management, Kütahya Health Sciences University, Germiyan Campus, Kütahya 43100, Türkiye; ruyameltem.sariyer@ksbu.edu.tr; 2 Reading School of Pharmacy, University of Reading, Whiteknights Campus, Reading RG6 6AD, UK; a.bye@reading.ac.uk; 3 Department of Genetics and Bioengineering, Rafet Kayış Engineering Faculty, Alanya Alaaddin Keykubat University, Alanya 07425, Türkiye; ilayda.eryilmaz@alanya.edu.tr; 4 Reading School of Biological Sciences, University of Reading, Whiteknights Campus, Reading RG6 6AS, UK; c.i.jones@reading.ac.uk; 5 School of Electronics and Computer Science, University of Southampton, Highfield Campus, Southampton SO17 1BJ, UK

**Keywords:** platelet aggregation, darkfield, light scattering, Raspberry Pi

## Abstract

**Highlights:**

**What are the main findings?**

- Platelet aggregation can be directly monitored by simple darkfield imaging of a microfluidic device.
- We present Dark-Pi, an open-source Raspberry Pi darkfield imaging setup.

**What are the implications of the main findings?**

- Measuring platelet activation and aggregation is important for thrombosis research and cardiovascular disease.
- Low-cost portable microfluidic systems offer potential for monitoring platelet function.

**Abstract:**

Measuring platelet function is important for patient stratification to judge bleeding vs. thrombotic risk and for research into antiplatelet drugs to prevent cardiovascular disease. Variability in platelet function is not fully understood, and large studies of inter-individual variation are making gradual progress using laboratory measurements, but rapid and high-performance hematological tests are also needed. We present here a novel microfluidic technology for platelet function analysis that images light scatter by platelets, using a low-cost, open-source, high-throughput and customizable darkfield imaging system called Dark-Pi. The hardware consists of a camera and a simple LED light source controlled by a Raspberry Pi, with 3D-printed parts. Using the Dark-Pi, platelet aggregation was imaged within adenosine 5′-diphosphate-loaded microcapillaries, revealing clearly visible patterns. This darkfield cellular light scatter approach was previously developed for bacterial cells, and here we adapted and optimized it for directly monitoring platelet aggregation. Capturing high-quality time-resolved images of platelets undergoing activation within microcapillaries allowed us to measure changes in light scattering in platelet-rich plasma that correspond with aggregation measured using conventional laboratory methods. This novel prototype system shows that this approach may have potential for use in large-scale studies of platelet function, combining simplicity with low-cost components and using a disposable dip-and-test microfluidic format.

## 1. Introduction

Platelets are small blood cells mostly produced by the bone marrow [1]. Since platelets play central roles in different processes such as hemostasis, inflammation, metastasis, wound closure and thrombosis, platelet function is important both diagnostically and to understand disease pathology [2]. Platelet function tests (PFTs) are diagnostic tests that check whether platelets are hyper- or hyporeactive [3]. PFTs must be performed rapidly after blood collection. Processes such as freezing, storing, and transporting platelet-containing samples over long distances must be avoided [3,4]. Thus, there is a need for point-of-care (POC) tests that can evaluate platelet function in a fast and simple manner, allowing high-throughput analysis with even small sample volumes, and can be used outside laboratories.

Various techniques have been developed to evaluate platelet aggregation, such as light transmission aggregometry (LTA), impedance platelet aggregometry, Lumi-aggregometry, and Plateletworks^®^ [5]. LTA, which remains the gold standard in clinical platelet diagnostics, has been used since the 1960s. LTA measures the transmission of light through a platelet-rich plasma (PRP) sample, which increases when platelets aggregate [6,7]. While the technology has become more compact and user-friendly over time, the fundamental approach has remained mostly the same [8]. Diagnosis of primary hemostatic defects therefore places significant demands on time, labor, expertise and blood samples. LTA application is primarily confined to specialized hematology laboratories [8]. Sets of guidelines play a crucial role in establishing a standardized approach to the methodology and interpretation of LTA testing [8]. Local compliance with published guidelines for LTA must be checked continuously [9,10]. There are extensive studies on the working principles of platelet activation by adding different agonists to PRP [11]. With developments in recent years, due to automation, LTA tests have become possible without requiring an expert. However, while saving personnel time, the reagent and consumable costs are higher than the traditional methods, and expert interpretation is still needed [8]. On the other hand, a modified form of LTA, known as Lumi-aggregometry, offers insights into platelet secretion simultaneously with assessments of platelet aggregation [8]. Many companies produce different kinds of POC tests to overcome the challenges of traditional methods for platelet measurements, including PFA-100, PFA-200 (Siemens), VerifyNow (Werfen), Plateletworks (Helena Laboratories), Impact R (DANED SA), Multiplate (Roche Diagnostics), TEG/ROTEM (TEG; Haemonetics Corporation/ROTEM), and Global Thrombosis Test (Thromboquest Ltd.) [12,13]. Despite a wealth of well-conducted studies, these techniques are still not recommended for routine clinical use [12,14]. Recent recommendations continue to emphasize that LTA is a specialist PRP-based assay with substantial pre-analytical, analytical and post-analytical requirements, and that standardization and accreditation remain important practical challenges [15].

Microfluidic systems offer significant potential benefits for hemostasis testing and diagnosis due to the requirement for blood volumes within the µL scale for platelet function, coagulation, thrombosis, adhesion dynamics, and clinical diagnostic measurements, without the requirement for perfusion or complicated mixing processes [16]. Several microfluidic devices have been developed to measure platelet aggregation. For example, platelet aggregation tests using microfluidic systems that consider multiple parameters were reported [17,18]. A portable device for measuring platelet aggregation impedance, called MICELI, which has the potential for POC platelet function testing, was presented [19]. Even an automated LTA test on a disk was introduced [5]. Recent reviews describe emerging microfluidic, optical and acoustic platelet-function technologies as complementary approaches that probe different aspects of platelet behavior rather than simple drop-in replacements for conventional LTA [20,21]. Microfluidic platelet-function tests offer small-volume and potentially rapid formats, but recent reviews highlight continuing challenges in flow-design optimization, scalable manufacturing, diagnostic reliability and clinical translation [21].

We previously developed a darkfield imaging system for visualizing bacteria in microfluidic devices [22]. Its technical advantages include its simplicity, affordability and ability to capture high-quality images. Here, we expanded the use of microfluidic technology to develop a prototype platform called Dark-Pi. We explored whether it is feasible to monitor and detect platelet aggregation by light scattering within microfluidic devices. We explored whether Dark-Pi was able to image platelet suspensions and monitor multiple samples simultaneously with time-lapse imaging, and whether this imaging was capable of detecting platelet aggregation. We established that platelet aggregation stimulated by Adenosine 5′-diphosphate (ADP) in microcapillary film (MCF) test strips results in detectable changes that can be digitally captured with a low-cost digital camera. We demonstrated at a proof-of-concept level that platelet aggregation can occur within microcapillaries and that we were able to detect and image this in the darkfield box with a very simple system.

## 2. Materials and Methods

### 2.1. Materials

HEPES-buffered saline (HBS) solution (10 mM HEPES, 150 mM NaCl, 1 mM MgSO_4_, 5 mM KCL, pH 7.4) and Tyrodes (vehicle) solution (134 mM NaCl, 2.9 mM KCl, 0.34 mM Na_2_HPO_4_.12H_2_O, 12 mM NaHCO_3_, 20 mM HEPES, 1 mM MgCl_2_, pH 7.3) were made using Fisher BioReagents components (Fisher Scientific UK Ltd., Loughborough, UK); ADP (product A2754), Tween 20 (product P1379), and polyvinyl alcohol (MW 146,000–186,000, >99% hydrolysed), were from Sigma Aldrich (Gillingham, UK). Cangrelor AR-C69931MX (4256J) was from The Medicines Company (Cambridge, MA, USA). Polyclonal Rabbit Anti-Human Fibrinogen/FITC (part number F011102-2) was from Agilent Technologies (Santa Clara, CA, USA). Green fluorescent membrane dye (DiOC6) was from Sigma Aldrich (Gillingham, UK). MCF containing 200 µm diameter capillaries and made from fluorinated ethylene propylene was obtained from Lamina Dielectric Ltd. (Billingshurst, UK).

### 2.2. Test Strip Production

The method of internally coating test strips with polyvinyl alcohol (PVOH), transforming the inner hydrophobic surface of microcapillaries to hydrophilic, was described previously [23,24,25]. PVOH-coated MCF strips were cut to 25 mm lengths. A 25 mm MCF strip containing ten 200 µm diameter capillaries has an approximate internal volume of 8 µL when fully filled. These PVOH-coated strips were divided into two parts to be used separately for different procedures, serving as control and test strips. Control strips were loaded with distilled water. Test strips were loaded with 10 mM ADP in water. The reagent loading protocol was previously described and involved freeze-drying reagents inside the test strips [25]. The control strips were dipped into PRP and platelet-poor plasma (PPP). The test strips were dipped into the PRP sample. These strips were monitored by darkfield imaging as described below (Figure S1). To ensure the quality of the test strips prior to the experiments, ADP-loaded and control strips were assessed for any crystallization using darkfield imaging (Figure S2).

### 2.3. Preparation of Blood Samples

Blood samples were collected from healthy donors, who had given informed consent in compliance with ethics approved by the University of Reading (UREC 20/20), into the 3.2% buffered sodium citrate blood collection tube (BD Vacutainer^®^; Becton, Dickinson and Company, Franklin Lakes, NJ, USA). After collection, whole blood was centrifuged (at 102× *g* for 20 min at 20 °C) to separate PRP. PPP was prepared by centrifugation of PRP (at 1413× *g* for 10 min at 20 °C).

### 2.4. Confocal Microscopy

Aggregation was imaged in MCF test strips after either pre-stimulating PRP with agonists in wells of a microtiter plate and then aspirating into MCF test strips or by aspirating PRP into MCF test strips pre-coated with agonist.

For pre-stimulation in microtitre plates, PRP or PPP was stimulated as described in figure legends either under static conditions or after shaking for 5 min at 1200 rpm using an orbital plate shaker (Quantifoil Micro Tools GmbH, Jena, Germany) prior to aspiration in MCF test strips. For stimulation within MCF test strips, test strips coated as described in figure legends were dipped into unstimulated PRP or PPP.

Representative capillaries from each test strip were imaged using a confocal A1R microscope (Nikon Corporation, Tokyo, Japan) using a 20× objective lens.

### 2.5. Light Transmission Aggregometry

**Figure 1:**
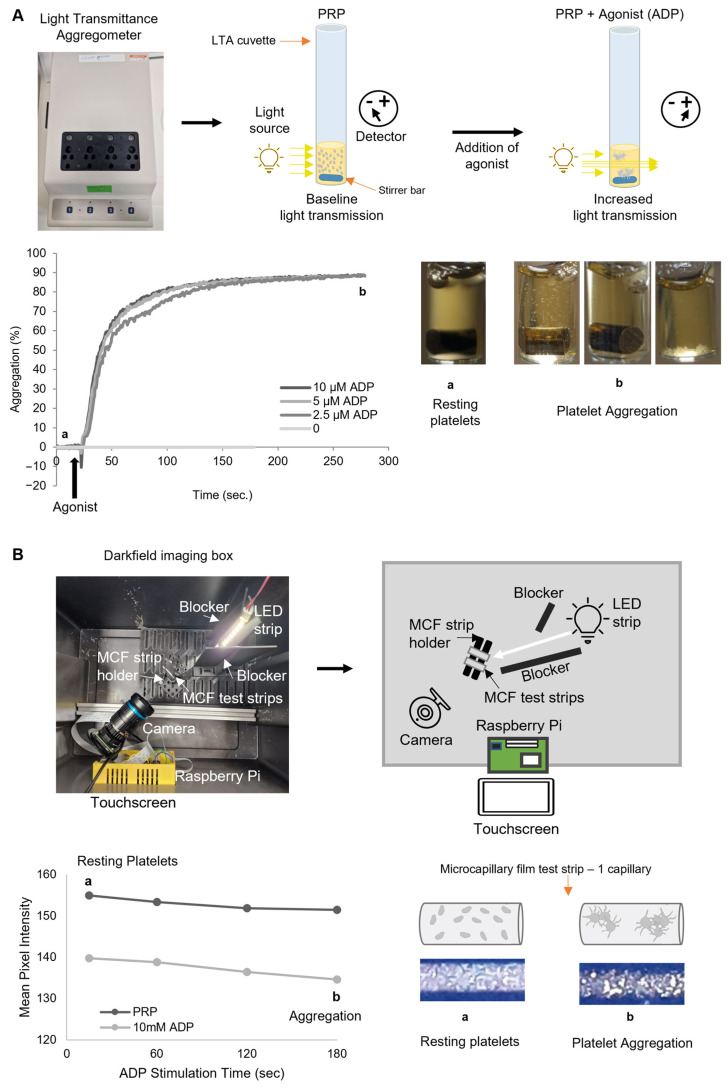
Light transmission aggregometry and darkfield microfluidic imaging of platelet aggregation. (**A**) LTA works on the principle that platelet aggregation increases light transmission. PRP and a stirrer bar are added to an LTA cuvette and placed in the device. With the addition of an agonist, the platelets begin to activate and change shape and then start to aggregate. When platelets aggregate, they absorb less light, resulting in increased light transmission, which is detected by the camera. At the end of the test, the percentage of light transmission or the percentage of platelet aggregation versus time is plotted graphically. (**B**) The darkfield imaging setup consists of 3D-printed parts (blockers, MCF rack, LED rack, etc.), a microdevice, a white LED light source, a Raspberry Pi HQ camera controlled by a Raspberry Pi and a touchscreen. The components are placed in a box, and only the touchscreen is mounted on the outer surface of the box. The microdevice is placed between the camera and the LED strip. The light is directed by blockers. In the darkfield box, platelets in the agonist-loaded microfluidic channels form aggregates and concentrated areas, resulting in a decrease in pixel intensity due to increased platelet aggregation. The test result is shown by a graphical representation of the mean pixel intensity over time.

Platelet aggregation was measured using a Helena AggRAM aggregometer (Helena Laboratories Corp., Beaumont, TX, USA) (Figure 1A). A cuvette was positioned between a light source and a detector. The aggregometer was calibrated against a PPP sample, and then aggregation of PRP was stimulated with 10 mM ADP at 37 °C while the sample was stirred at 1200 rpm using a magnetic stir bar. Aggregation evoked by 10 mM ADP was also measured in the absence of stirring. Responses were recorded for 5 min. The addition of an agonist induces platelet aggregation, increasing light transmission, which is measured by a detector.

### 2.6. Flow Cytometry Assay

Flow cytometry analysis was conducted on samples extracted from the ADP-loaded test strips and control strips. Samples from the microcapillaries were aspirated using a syringe and added to flow cytometry tubes. Despite efforts to minimize activation, our method could not prevent platelet activation. HBS, 250 mM EDTA, PRP, and anti-fibrinogen were added to the flow cytometry tube, then incubated in the dark for 5 min and subsequently fixed with 0.2% formyl saline. The analysis was run with the following limits: 1 min medium speed, and threshold from 80,000 to 20,000. The gate was drawn for percent positive. For samples in capillaries, for the control, HBS, aspirated PRP, and anti-fibrinogen were added to the flow cytometry tube. Additionally, for ADP stimulation, HBS, aspirated PRP with ADP, and anti-fibrinogen were added to another flow cytometry tube. Then these tubes were also incubated and fixed. Analysis was run with the following limits: 10,000 events, medium speed, and threshold 20,000. The assay was repeated 4 times with different healthy donors.

Fluorescein isothiocyanate-conjugated (FITC) anti-fibrinogen antibody (Dako; Agilent Technologies LDA UK Limited, Stockport, UK) binding and percent platelet aggregation following stimulation with ADP were measured using an Accuri C6 (Becton, Dickinson U.K. Limited, Wokingham, UK). FITC-labeled anti-fibrinogen antibodies show fibrinogen binding in platelets. By measuring this fluorescent signal with flow cytometry, the percentage of platelet aggregation is determined. Median fluorescence intensity data was saved for each condition. The results were compared with the aspirated samples, PRP and PRP with ADP, and the PRP sample in the microwell plate.

### 2.7. Darkfield Microfluidic Imaging System and Optimization

A Raspberry Pi high-quality (HQ) camera and high-resolution lenses (Raspberry Pi (Trading) Limited, Cambridge, UK) were employed to observe platelets and smaller particles (approximately 2 µm) [26] with greater detail and gain insights into aggregation. A Raspberry Pi model 4 (4 Gb RAM model) and a Raspberry Pi 7′ touchscreen (Raspberry Pi (Trading) Limited, Cambridge, UK) were added to the design, as well as an HQ camera (Figure 1B).

The position of the light source, camera, and sample were adjusted to define the most effective parameters for detecting platelet aggregation. A number of parameters were tested to determine optimum conditions; these included adjusting LED and sample distances, adjusting camera and sample distances, and using different test strip lengths. Other parameters included using black and gray 3D-printed parts and box color and testing different lenses. The tested parameters are detailed in Table 1. Additional photographs illustrating all components, including the MCF test strips, strip holder, and touchscreen, are provided in Figure S1, alongside example images of raw darkfield views. Notably, the overall light scatter intensity varied by position, so analysis was always performed at the same location along the test strip.

### 2.8. Darkfield Imaging of Platelet Aggregation

The strips were gently cleaned with a microscope lens cleaning wipe to remove any dust that could affect the imaging, as dust and particulates can strongly scatter light, giving unwanted noise in the images. In this study, we explored various experimental conditions to assess whether platelet aggregation can be visualized within capillaries using the darkfield imaging box system. As an additional control, to determine whether cangrelor blocks ADP-induced activation and aggregation of platelets within the microcapillaries, ADP-loaded test strips were dipped into samples of PRP in the presence of 10 mM cangrelor in water. Strips dipped into the samples were placed in the system. For dipping, approximately 25 µL of sample was placed in the well to ensure contact with the bottom of the strip and thereby ensure reliable capillary uptake. The first recorded image available for analysis was acquired 10 s after dipping; no image was acquired before this timepoint. Time-lapse images were captured every 10 s for 3 min. The images were then transferred to a computer for image analysis.

### 2.9. Data Analysis

Images were analyzed using ImageJ software (version 1.54m) [27] by first drawing a 200 × 28-pixel region of interest (ROI) around each capillary and then extracting mean pixel intensity values. Preliminary checks using left, central and right ROI positions showed that ROI position affected absolute mean pixel intensity values, but the relative separation between PRP control and ADP-loaded strips was preserved. Therefore, for the main analysis, a consistent ROI size was used, and the ROI was placed on the right side of each capillary, where illumination was more homogeneous and light scattering intensity was higher, likely due to the smaller scatter angle from the LED light. Histogram analysis was then used to quantify changes in platelet aggregation for each capillary. Mean pixel intensity values were plotted against time to construct aggregation time-course plots. No full-field illumination correction was applied in this proof-of-concept study; therefore, capillary-to-capillary variability, illumination variation and ROI-position sensitivity remain significant limitations of the current prototype that should be addressed in future automated analysis.

Statistical analysis was performed on the data obtained from flow cytometry. Statistical analysis was performed using GraphPad Prism version 10.0.0 (GraphPad Software, Boston, MA, USA). The data was first tested for normal distribution. The normality of the data was examined using the Shapiro–Wilk test. Then, based on the results, one-way ANOVA and the Friedman test were applied. To examine the differences between the groups in more detail, Tukey’s Honestly Significant Difference (HSD) test was applied.

## 3. Results

### 3.1. Effect of Stirring on ADP Stimulation

**Figure 2:**
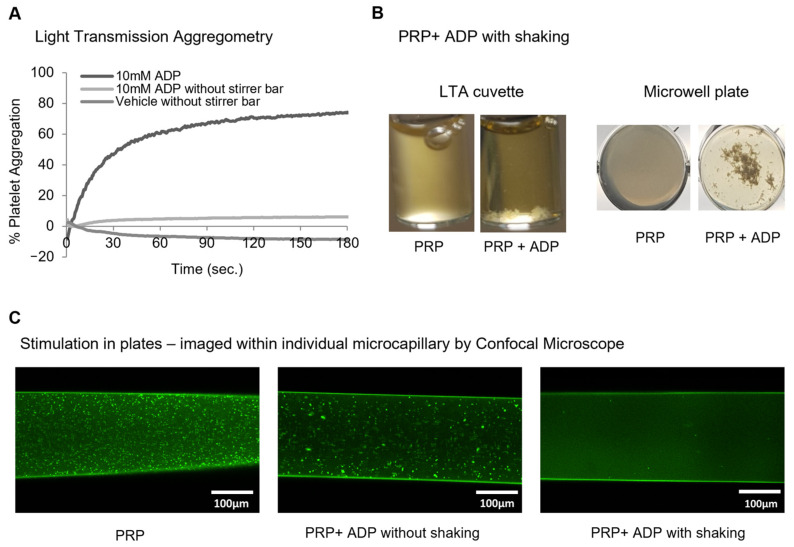
ADP stimulation in LTA cuvette and in microwell plates with and without shaking. All panels show qualitative observations from the same biological donor (*n* = 1) and were used to illustrate platelet aggregation behavior under stirred/shaken and non-stirred conditions. (**A**) LTA test was performed by adding 10 mM ADP into the PRP with and without a stirrer bar, and a vehicle control (Tyrode’s: standard buffer) without a stirrer bar. Platelet aggregation was monitored for 180 s. The 6.27% aggregation was observed in the ADP-stimulated PRP sample even without stirring. (**B**) In the presence of ADP, PRP forms large visible aggregates in the LTA test cuvette when mixed with a stirrer bar and in microwell plates after shaking with a plate shaker. (**C**) Images of one representative selected capillary were obtained by confocal microscopy in the presence of fluorescent dye (DiOC6) in the test strips. In the presence of ADP, PRP formed large, visible aggregates that settled in the microwell plates when shaken with a plate shaker, resulting in no detectable platelets in the capillary. These qualitative observations were obtained from one donor and are used to illustrate the practical limitation of transferring stirred or pre-mixed samples into the MCF strip format.

LTA uses stirring to promote platelet aggregation; however, it is not possible to incorporate stirring into the microfluidic capillaries. Stimulating platelet aggregation prior to loading PRP into microfluidic capillaries is not possible due to the formation of large aggregates that are too big to enter the microcapillaries, thus complicating the measurement of aggregation within the strips. Therefore, we initially investigated whether platelet aggregation occurred in the absence of stirring by comparing LTA and MCF strips. In LTA, PRP with a 10 mM ADP sample, with the addition of a stirring bar, reached almost 80% platelet aggregation, as expected (Figure 2A). On the other hand, the maximum aggregation of the PRP with a 10 mM ADP sample without an added stirrer bar was measured as 6.27%, indicating that we could measure a small amount of platelet aggregation even without stirring. To observe the natural state of platelets and how they behave without being exposed to any agonist during the test, we added Tyrode’s standard buffer (vehicle) to PRP, and as expected, the maximum aggregation recorded was 0% of the starting signal. Performing the experiment without the use of a stirrer bar does introduce a further variation. The absence of stirring leads to slower mixing of the reactants, which affects the zeroing of the aggregometry measurement. Nevertheless, detectable changes in aggregation are still measurable.

In the presence of ADP, PRP formed large visible aggregates that sedimented in LTA test cuvettes when mixed with a stirrer bar or within microwell plates shaken with a plate shaker (Figure 2B). Having observed an aggregation of 6.27% in LTA without stirring in cuvettes, we next transferred these non-stirring conditions into a microplate format to allow interfacing with microcapillary test strips and allow us to observe if aggregation also occurs within the microcapillaries. After the addition of fluorescent dye (DiOC6) to these samples, microcapillary test strips were dipped, and the samples were viewed under a confocal microscope (Figure 2C). While individual dispersed platelets were observed in the control unstimulated PRP sample, small platelet aggregates were clearly detected when the MCF strip was dipped in the PRP sample in the presence of ADP without stirring. In contrast, when microwell plates were shaken with ADP, no platelets were observed in the capillary, indicating aggregates were too large to be drawn up. Indeed, the aggregates were clearly visible in the shaken microwell (Figure 2B).

### 3.2. The Dark-Pi Approach: Detection of Platelet Aggregation Stimulated Within Microcapillaries

**Figure 3:**
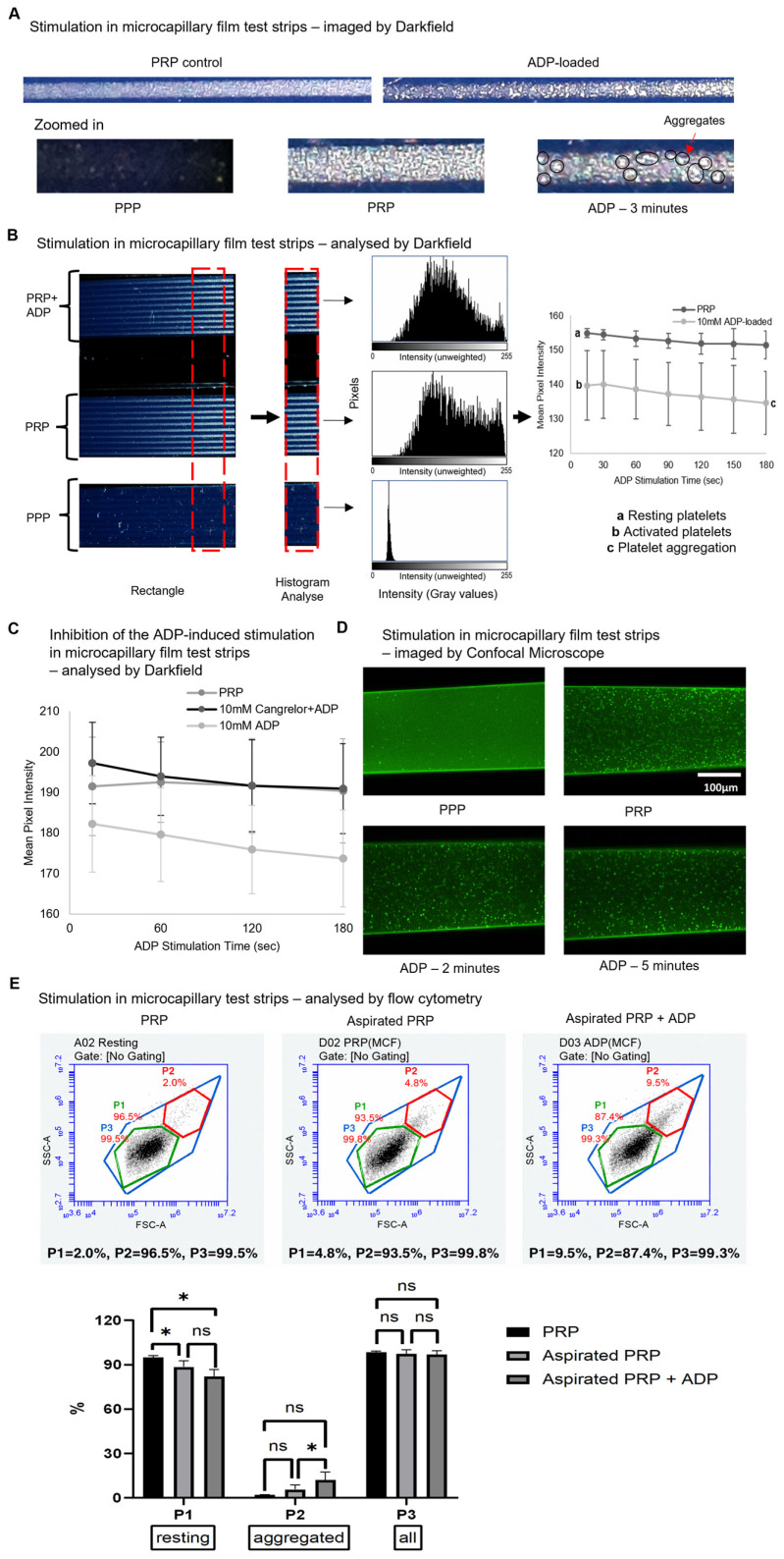
Stimulus within the MCF activates platelets, and platelet aggregation can be visualized using the darkfield system. (**A**) Images of ADP-loaded test strips show visible changes compared to PRP control strips. A more detailed zoomed image is shown by selecting one capillary each for PPP, PRP and PRP with ADP. (**B**) For image analysis of stimulation within MCF test strips, a rectangular ROI was selected in ImageJ, and histogram analysis was applied separately for each capillary. Representative ImageJ histogram outputs are shown to illustrate the analysis workflow; the *x*-axis represents unweighted pixel intensity from 0 to 255, and the *y*-axis represents pixel count. Changes in mean pixel intensity over time are shown for 15, 30, 60, 90, 120, 150 and 180 s. The plotted values represent the average pixel intensity from three biological donors (*n* = 3), with ten capillaries analyzed per condition for each donor. Error bars show the standard deviation of the mean. Mean pixel intensity decreased over time in the ADP-loaded strips. (**C**) Effect of cangrelor on ADP-induced stimulation in MCF test strips. The data show the mean pixel intensity from three biological donors (*n* = 3), with each biological replicate comprising ten technical capillary replicates for each condition. Error bars indicate the standard deviation of the mean. (**D**) Representative confocal image of one selected capillary showing stimulation in MCF test strips. (**E**) Flow cytometry analysis showed increased aggregates in P2 and decreased single platelets in P1, consistent with shifting individual platelets from the P1 region to the P2 region by forming aggregates (*n* = 4 biological donors). * indicates a statistically significant difference between the connected groups (*p* < 0.05); ns, not significant.

Having established that small changes in platelet aggregation can be detected in the absence of stirring, we tested if the Dark-Pi imaging prototype was capable of directly detecting platelet aggregation during stimulation within microcapillaries. MCF control and test strips were dipped into samples and placed in the darkfield box, and time-lapse images were taken for 3 min. A clearly visible difference was observed between the strips between PPP, PRP control and PRP taken up into ADP-loaded strips (Figure 3A). Images of the PPP strip featured dark capillaries due to the absence of light-scattering particles in the sample. Homogeneously distributed bright light scattering patterns were observed in the PRP control strip due to the presence of evenly distributed single platelets. Bright clusters were observed in the ADP-loaded strip, which corresponded to the formation of platelet aggregates. The darkfield system successfully differentiated between unactivated and activated states, providing clear visual distinctions between resting and aggregated platelets (Figure S3). Representative raw darkfield image sequences are shown in Figure S4, and stimulation dynamics were quantified in Figure S5. This provides us with initial proof-of-concept evidence that it is possible to directly capture platelet activation using a simple, low-magnification digital imaging system.

In Figure 3B, analysis of intensity changes was performed using three biological donors by assessing intensity histograms, with ten capillaries analyzed per condition for each donor, providing 10 replicate measurements for three biological replicates. Mean pixel intensity values were plotted at 15, 30, 60, 90, 120, 150 and 180 s after dipping to show the time course of platelet stimulation. As can be seen from the histogram analysis applied to each capillary in this selected area (Figure 3B), a mean pixel intensity value of 32.87 ± 2.73 was measured as the average of three donors for the microcapillaries dipped in PPP. This shows that this image has low pixel intensity—as expected in the absence of light-scattering platelets. In contrast, the mean pixel intensity for the control strips dipped in PRP was consistently higher, with a mean intensity of 152.9. The ADP-loaded test strips showed lower pixel intensities than control strips, with a mean intensity of 137.4. PRP mean pixel intensity remained relatively stable over the analyzed time course, suggesting no change in light scattering without stimulation. In the ADP-loaded test strips, mean pixel intensity gradually decreased over time from 139.7 to 134.6, suggesting a continuous change in light scatter while the platelets respond to ADP. These data were obtained from three biological donors, with ten capillaries analyzed for each condition for each donor. Individual donor-level traces and capillary-to-capillary variability are shown in Figure S7. ADP-loaded MCF strips showed consistently lower mean pixel intensity than paired PRP control strips across the three donors over the analyzed time course (Figure S7a). Capillary-to-capillary variability, calculated as CV from ten capillaries per condition, remained approximately 5–7% across the analyzed timepoints (Figure S7b), providing a quantitative estimate of technical variability associated with capillary position, illumination differences and ROI-based analysis. The capillary-level values used for this analysis are provided in Table S1. Given the variability of platelet responses in individuals, a fully developed test would require more extensive validation across larger populations of donors to confirm these initial observations.

To demonstrate that these changes in light scatter were the result of ADP-induced platelet aggregation and not an artifact, we blocked platelet ADP receptors with the P2Y12 receptor antagonist cangrelor [28]. Figure 3C shows that cangrelor effectively inhibits platelet activation within the MCF test strips.

We then sought further validation of our findings by imaging MCF strips using confocal fluorescence microscopy, following stimulation within the ADP-loaded test strips. We detected individual platelets in the PRP sample, small aggregates in the ADP sample and an absence of platelets in the PPP sample (Figure 3D).

To determine to what extent platelet aggregation had occurred within the capillary, we used flow cytometry to quantify changes in the number of single platelets and small platelet aggregates. The P1 region includes individual, non-aggregated platelets, P2 covers aggregated platelets consisting of two or more adjoined platelets, and P3 covers total platelets (Figure 3E). Similar results were obtained across four biological donors. The percentages of platelets within each of these gates are shown on the flow cytometry charts for one representative donor. The data was obtained from four different healthy donors. The average P1% values for PRP, PRP aspirated from the control strip, and PRP aspirated from the ADP-loaded test strip were 95.05 ± 1.16, 88.58 ± 4.05, and 82.06 ± 4.70, respectively. For the same samples, the average P2% values were 1.98 ± 0.05, 5.65 ± 3.00 and 12.10 ± 5.35, and the average P3% values were 98.60 ± 0.60, 97.32 ± 2.78 and 96.92 ± 2.54, respectively. The P1% values show a decreasing trend from the PRP sample towards aspirated PRP from the ADP-loaded test strips, which may be attributed to ADP increasing aggregation between platelets.

The presence of ADP causes platelets to aggregate, and fewer individual platelets were detected in the P1 region. P2% values increased, suggesting that ADP promotes platelet aggregation. The presence of ADP would be expected to increase platelet adhesion to each other, leading to more aggregate formation in the P2 region. P3% values usually remain almost constant or show a slight decrease because the P3 region represents the total platelets, and these values usually do not change. Therefore, we can conclude that the main reason for the changes in the P1 and P2 regions is that ADP affects platelet behavior and increases aggregation.

Individual and aggregated platelet percentages in the P1, P2, and P3 regions obtained from PRP and two other samples aspirated from capillaries were evaluated using an appropriate test to see whether there was a significant difference between the groups. Our one-way ANOVA analysis revealed a significant difference in P1 values between different groups (F (1.487, 4.461) = [18.77], *p* = 0.0078). When we used Tukey’s HSD Test for a more detailed analysis, we found significant differences, especially between PRP and aspirated PRP (*p* = 0.0437, 95% CI = [0.3275, 12.60]) and between PRP and aspirated PRP with ADP (*p* = 0.0184, 95% CI = [4.026, 21.96]). However, no significant difference was observed between aspirated PRP and aspirated PRP with ADP (*p* = 0.1658). Similarly, we performed a one-way ANOVA analysis, which showed a significant difference between groups in P2 values (F (1.095, 3.284) = [14.21], *p* = 0.0274). Following that, Tukey’s HSD Test showed that there was a significant difference between aspirated PRP and aspirated PRP with ADP (*p* = 0.0352, 95% CI = [−12.09, −0.8058]). However, no significant differences were observed between PRP and aspirated PRP (*p* = 0.1729) or between PRP and aspirated PRP with ADP (*p* = 0.0632). A Friedman test revealed that there was no statistically significant difference in P3 value between the groups (*p* = 0.9306).

While the standard assay was completed in 3 min, an extended observation was also performed to evaluate prolonged aggregation dynamics. Continuous monitoring of platelet stimulation within the MCF test strips for up to 10 min showed that the system could successfully track these events over a longer duration. Time-lapse images of a selected capillary acquired every minute for 10 min suggested that sedimentation started gradually after the third minute (Figure S6). This may suggest that slower platelet changes occur in the absence of mixing, and a longer analysis may be required in microfluidic devices that do not include a mixing process.

## 4. Discussion

In this study, we present proof-of-concept for a microfluidic platform that enables direct digital imaging detection of platelet aggregation within microcapillaries, called Dark-Pi. We showed that platelet aggregation induced by ADP can occur within microcapillaries (in the absence of mixing) and that we can detect and visualize this in a very simple system, comprising darkfield imaging by a CMOS digital camera. In the late 1990s, a platelet aggregometer method was developed based on the principle of light scattering [29,30]. This method involves sending laser beams to platelet aggregates, which produce light scattering, which is then detected [29,31]. The light scattering provides information about the size and number of aggregates. Following this development, a platelet aggregometer (PA-100, KOWA) was used to determine platelet aggregate size [32], and following this, a simple method to assess the presence of native microaggregates was developed (PA-200; Kowa, Tsukuba, Japan) [33]. These platelet aggregometers use a particle counting method based on laser light scattering [32,33,34]. In the early 2000s, PRP aggregation was assessed simultaneously by the maximal percentage decrease in optical density in the conventional aggregometry method and by light scattering intensity-based aggregometry (AG-10 aggregometer, Kowa Co. Ltd., Tokyo, Japan) [35]. In the following years, platelet aggregation continued to be measured by different laser-light scattering techniques [36]. As a result of some limitations in traditional platelet aggregation methods, researchers have aimed to develop new devices by putting forward new ideas that can eliminate these limitations. Light scattering remains the most widely used method to monitor the activation and aggregation of platelets. Many studies have explored laser light scattering methods since the 1990s. Today’s microfluidic technology adds a different dimension to this situation and enables the development of novel, simple and affordable platelet aggregation tests. Laser light scattering methods in the literature are based on the fact that the intensity of the scattered light emitted by a particle increases in direct proportion to the square of the diameter of the particle [31]. In these methods, the sample, which is generally kept in a cuvette, is stirred with a magnetic stirrer bar. When a diode laser beam passes through a limited area of a sample, the intensity of the light scattered by the particles is recorded. The received light signals are digitized by a converter and processed by a computer. Microfluidic devices can be used to stimulate small amounts of whole blood or PRP. Imaging methods with a wider field of view but lower resolution may be needed because multiple channels might be observed. In the microfluidic method we have newly developed based on light scattering to measure platelet aggregation, we use the method of directly dipping stimuli-loaded strips, recording and analyzing the obtained images, without any mixing and without the need for an additional process. By using a simple LED strip instead of a laser beam, we record the light scattering with a Raspberry Pi camera without the need for a detector and then obtain light intensity data from the images transferred to the computer by histogram analysis. Here, we present a method that can detect changes in platelet aggregation following stimulation within microcapillaries with a lower resolution and label-free light scattering imaging system.

One significant consideration is the absence of mixing within capillaries. LTA, laser light-scattering aggregometers and many microfluidic thrombus assays use different mixing, stirring, shear or flow conditions that rapidly drive platelet aggregation. In the absence of stirring or shear within capillaries, direct comparison between standard methods and our Dark-Pi prototype is not straightforward. Table 2 compares operating principles, practical strengths and current limitations between these systems, rather than assessing analytical equivalence between these methods. A recent laser-speckle microfluidic approach measured platelet-function-related changes through speckle size and contrast using a laser, microscope objective and high-speed CMOS camera, illustrating the growing interest in optical microfluidic platelet readouts while contrasting with our simpler LED/Raspberry Pi hardware used here [37]. Recent advanced microfluidic assays can also profile thrombus formation under stenosis-like high-shear and high-gradient flow, reinforcing that microfluidic platelet platforms often address different biological questions from stirred cuvette aggregometry [38]. While LTA indirectly measures platelet aggregation by monitoring changes in light transmission, microcapillary-based imaging systems directly visualize platelet aggregation within microcapillaries. In LTA, the addition of an agonist induces platelet aggregation, leading to changes in light transmission [39], whereas in microcapillary-based imaging systems, platelet aggregation is visualized as concentrated regions within agonist-loaded microcapillaries and measured as light scattering, indicating platelet aggregation. LTA plots the percentage of light transmission or platelet aggregation against time, while microcapillary-based imaging systems use histogram analysis to track changes in pixel intensity over time. Both methods facilitate the characterization of platelet states, including resting, activated and aggregated platelets. Although LTA is a widely used method, it requires a large sample volume and is also expensive and technically demanding, requiring careful control and application by skilled personnel in specialized laboratories [39,40]. Therefore, the use of cheaper and easier-to-use microfluidic platforms that require less sample volume to measure platelet aggregation may become more widely available and become a standardized method among laboratories.

As seen in Figure 2B, shaking PRP with ADP before dipping caused large aggregates to form in the microwell. These large pre-formed aggregates were visibly sedimented and were not observed entering the microcapillaries. This observation reflects the difference between aggregation in bulk PRP during microplate mixing vs. the intended operating mode of the “dip-and-test” Dark-Pi assay that lacks external mixing or shaking. In stirred or shaken bulk PRP, platelet aggregates can grow before the sample enters the microcapillaries, and these large pre-formed aggregates may sediment in the microwell or be physically excluded during capillary uptake. As the microcapillaries used in this study were 200 µm in diameter, the system is expected to detect aggregates that can enter or form within the capillaries, but it does not define a calibrated aggregate-size detection range. Detection of larger aggregates in clinical samples will therefore require further validation, and introducing mixing or other fluidic features may also be necessary to explore the formation of larger aggregates if these are found to be clinically relevant.

In aggregometers, magnetic stir bars are used throughout the aggregation process to continuously mix PRP samples with an agonist at a constant speed [47]. Many microfluidic platelet aggregation tests instead use different physical conditions, including shear-induced platelet aggregation [43,45,46,48,49] or diffusion-based mixing [44]. In this study, we introduce a microfluidic platelet aggregation test in which there is no external mixing, shaking or agitation after dipping. The aim of the Dark-Pi format is not to transfer and size pre-formed bulk aggregates but to monitor platelet stimulation and aggregate formation directly within the disposable MCF test strip after dipping. In this format, ADP is pre-loaded in the MCF test strip and PRP is drawn into the microcapillaries by capillary rise. The short diffusion distance within the microcapillaries allows local interaction between the agonist and platelets, while the confined capillary geometry creates an unstirred microfluidic environment in which capillary flow and wall shear may also contribute to platelet stimulation. Although lack of mixing may reduce the size of aggregates formed compared to mixed assays, the fluidic simplicity—and associated low device complexity—may provide a trade-off if clinically relevant measurements can still be detected.

When PRP and ADP were shaken in a plate shaker before the strips were dipped into the sample, large aggregates formed in the microwell before capillary uptake. These aggregates were too large to enter the MCF efficiently, making it difficult to observe platelet aggregation within the microcapillaries. However, when the shaking step was omitted and ADP-loaded strips were dipped directly into PRP, platelet aggregates could be observed within the microcapillaries, as shown in Figure 2C. In our previous study [25], we found that the early phases of capillary rise in vertical microfluidic strips yielded average superficial velocities ranging from 10 to 30 mm s^−1^ (corresponding to theoretical wall shear rates between 100 and 600 s^−1^). Therefore, even without an external mixer, capillary rise can generate flow and wall shear conditions that may contribute to platelet stimulation within the MCF. This distinguishes Dark-Pi from conventional stirred cuvette aggregometry and helps explain why direct numerical equivalence with LTA is not expected. The confocal images therefore support the intended operating mode of the assay by showing platelet aggregation still occurs within the MCF under the dip-and-test condition, rather than after external bulk mixing. Together, these findings suggest that—as expected—shaking can strongly alter platelet behavior before capillary uptake, whereas Dark-Pi is designed to monitor aggregation that develops within the microfluidic strip itself; thus, detection of small aggregates within the capillary and without shaking remains an encouraging observation, with further study justified to explore how this in-capillary aggregation compares with bulk mixed aggregation in LTA.

Darkfield image analysis allows the evaluation of light scatter intensity by plotting the distribution of gray values across a selected area of the MCF test strip using a histogram plot. The higher light intensity of PRP indicates that this sample reflects more light and therefore contains more platelets. The low pixel intensity of PRP in ADP-loaded strips caused ADP to increase aggregation between platelets, leading to less light scattering and consequently recording a lower intensity value. In Figure 3B, we looked at the endpoint data after 180 s, and there was a difference of 17 intensity units. The effect of ADP on PRP might be quantified and used to determine the degree of aggregation. Additionally, the decrease in pixel intensity over time in samples with ADP can be interpreted as the continuous aggregation of platelets. As platelets aggregate, less light is scattered, causing the sample to become more opaque; this was recorded as low pixel intensity.

The ADP P2Y12 receptor subtype plays a crucial role in the processes of platelet activation and amplification. The inhibition of platelet activation by cangrelor (Figure 3C) also demonstrates that the microfluidics test is sensitive to commonly used antiplatelet medications and may have potential for monitoring the efficacy of antithrombotic therapies.

In traditional platelet function tests, obtaining individual resting platelets requires gentle handling to prevent their activation prior to analysis. However, due to the nature of our method and the necessity to handle samples differently, even our aspirated PRP showed signs of activation. Despite attempts to minimize activation, our flow cytometry analysis revealed increased aggregation, particularly in PRP aspirated from ADP-loaded test strips. Statistical analysis confirmed no significant loss of platelets, indicating consistent sample integrity. Notably, there was a shift towards increased aggregates and decreased single platelets within the capillary, affirming our ability to detect platelet aggregation using our MCF test strips. The authors developed a fully automated centrifugal microfluidic disk capable of performing LTA with small-volume (<1 mL) whole-blood samples [5]. This disk automates the entire LTA process within 25 min, including the preparation of PRP and PPP from whole blood, measurement of precise volumes, mixing with agonists, and detection of light transmission. On the other hand, the developed darkfield imaging platform aims to visualize platelet aggregation within microcapillaries containing 10 parallel capillaries, each capable of being loaded with a different agonist, unlike LTA, based on light scattering. Depending on the camera selection, 2 to 5 MCF strips can be added to the system, enabling the simultaneous analysis of 20 to 50 different conditions. Both systems have demonstrated effectiveness in evaluating platelet aggregation. In particular, both methods have been successful in characterizing platelet states and evaluating the effects of agonists. Thus, the LTA disk system and darkfield microfluidic system offer different but complementary approaches to evaluating platelet aggregation. Both methods can play an important role in clinical practice and provide valuable information to researchers.

## 5. Conclusions

Our darkfield imaging technique utilizes microfluidic strips with open-source hardware and software, illustrating that it is possible to detect platelet aggregation within the MCF test strips. Although agitation of the platelet sample, such as stirring or shaking, is not possible in microcapillaries, even small changes in platelet aggregation under static conditions were detectable using our highly sensitive darkfield imaging technology. Capturing high-quality and time-lapse images of platelet aggregation with our system enables measurement of time-dependent changes in aggregation based on light scattering. The simplicity of the system and the fact that it is a rapid test have the potential to bring significant benefits for platelet function research and diagnostics without the need for expensive equipment and expert personnel.

## Figures and Tables

**Table 1:** Tested parameters and optimal conditions.

Parameter	Range Tested	Optimal Condition	Comment
Camera lens type	35 mm 10 MP Telephoto Lens, 16 mm 10 MP Telephoto Lens	Both	The wide-angle lens is designed to observe 5 MCF test strips, while the telephoto lens allows for a closer and more detailed view of 2 test strips.
Distance between LED and sample	100 mm, 110 mm, 130 mm, 170 mm	170 mm (for 35 mm lens), 110 mm (for 16 mm lens)	Lens type affects the distance of illumination from the sample.
Distance between camera and sample	35 mm, 55 mm, 100mm, 130 mm	130 mm (for 35 mm lens) 55 mm (for 16 mm lens)	Lens type and distance affect field of view and number of test strips.
3D parts and box color	Black, gray	Black	Gray background and parts caused unwanted shadows on images.
MCF strip length	17 mm, 25 mm, 60 mm	25 mm	While the 17 mm test strip creates difficulty in placing the ladder, the 25 mm strip extends slightly from the ladder, making it easier to hold and place it. Longer strip lengths do not make it possible to observe platelets because the light reflected from the ladder brightens the image.

**Table 2:** Comparison of Dark-Pi with conventional and microfluidic platelet function testing technologies.

Technology	Typical Sample/Format	Mixing or Flow Condition	Readout	Strength	Current Limitation
**Light transmission aggregometry (LTA)** [6,8,15,40]	PRP in cuvette	Stirred; agonist added in cuvette	Light transmission	Established reference method with current guidance	Specialist workflow; sensitive to pre-analytical/analytical variation; direct comparison to non-stirred capillary assays is limited
**Flow cytometry** [41,42]	PRP or whole blood aliquots	Tube-based aliquots; no MCF format	Fluorescent marker/gated platelet populations	Cellular-level activation/aggregation information	Label-dependent; specialist equipment; sample handling may activate platelets
**Laser light scattering aggregometry** [29,30,31,32,36]	PRP/cuvette	Usually stirred	Scattered light/aggregate size/count	Measures aggregate number and size	Dedicated laser/detector instrumentation; not disposable MCF format
**Optical/speckle microfluidic assay** [37]	Whole blood or plate-let-poor/whole blood in micro-channel	Microchannel flow	Laser speckle size/contrast	Recent optical microfluidic readout; small sample volume	Requires laser/objective/high-speed sCMOS/PDMS device; still requires validation
**Microfluidic platelet/thrombus assays** [16,18,21,24,25,38,43,44,45,46]	Whole blood or PRP in microfluidic channels	Flow, shear, stenosis-like gradients or diffusion-based mixing	Imaging, impedance or thrombus formation	Probes physiologically relevant flow/shear or microenvironment-specific behavior	Often needs pumps, coatings or complex chip design; translation and standardization challenges remain
**Automated LTA on disk** [5]	Whole blood processed on centrifugal disk	Automated mixing and optical detection	Light transmission	Automates parts of LTA workflow	Still based on LTA principle; specialized disk platform
**Dark-Pi**	PRP in disposable MCF test strips	No active mixing after dipping; capillary uptake	Darkfield pixel intensity/time-lapse images	Low-cost, open-source, label-free, parallel MCF strip imaging	Feasibility stage; small donor cohort; no calibrated aggregate-size range; needs larger donor and clinical validation

## Data Availability

The data presented in this study are available within the article and Supplementary Material.

## Supplementary Materials

The following supporting information can be downloaded at: https://www.mdpi.com/article/10.3390/s26144326/s1, Figure S1: Dark-Pi setup and MCF test strip positioning; Figure S2. Assessment of crystallization in ADP-loaded and control strips using Darkfield imaging; Figure S3. Example images of capillaries with resting vs. aggregated platelets; Figure S4. Representative raw time-lapse darkfield images; Figure S5. Platelet stimulation within ADP-loaded microcapillary film test strips; Figure S6. Platelet stimulation in microcapillary film test strips for 10 minutes; Figure S7. Donor-level and capillary-level Dark-Pi intensity data used for platelet aggregation analysis; Table S1: Capillary-level Dark-Pi mean pixel intensity data from seven analyzed time points.

## Abbreviations

The following abbreviations are used in this manuscript:

PFTs	Platelet Function Tests
POC	Point-of-Care
LTA	Light Transmission Aggregometry
PRP	Platelet-Rich Plasma
ADP	Adenosine 5′-diphosphate
MCF	Microcapillary Film
HBS	HEPES Buffered Saline
PVOH	Polyvinyl Alcohol
PPP	Platelet-Poor Plasma
FITC	Fluorescein Isothiocyanate-Conjugated
HQ	High Quality
ROI	Region of Interest
HSD	Honestly Significant Difference
