# Supplementary material for: Dark-Pi Imaging System Permits Open-Source Label-Free Microfluidic Monitoring of Platelet Aggregation by Cellular Light Scattering
Source: Sensors (Basel). 2026 Jul 8;26(14):4326. doi: 10.3390/s26144326 (PMC13418586; doi:10.3390/s26144326)
Supplement: Supplementary file 1 [file sensors-26-04326-s001.zip › sensors-4343886-supplementary.pdf]

sensors

MDPI

Article

Dark-Pi imaging system permits open-source label-free microfluidic monitoring of platelet aggregation by cellular light scattering

Supplementary Figures and Tables

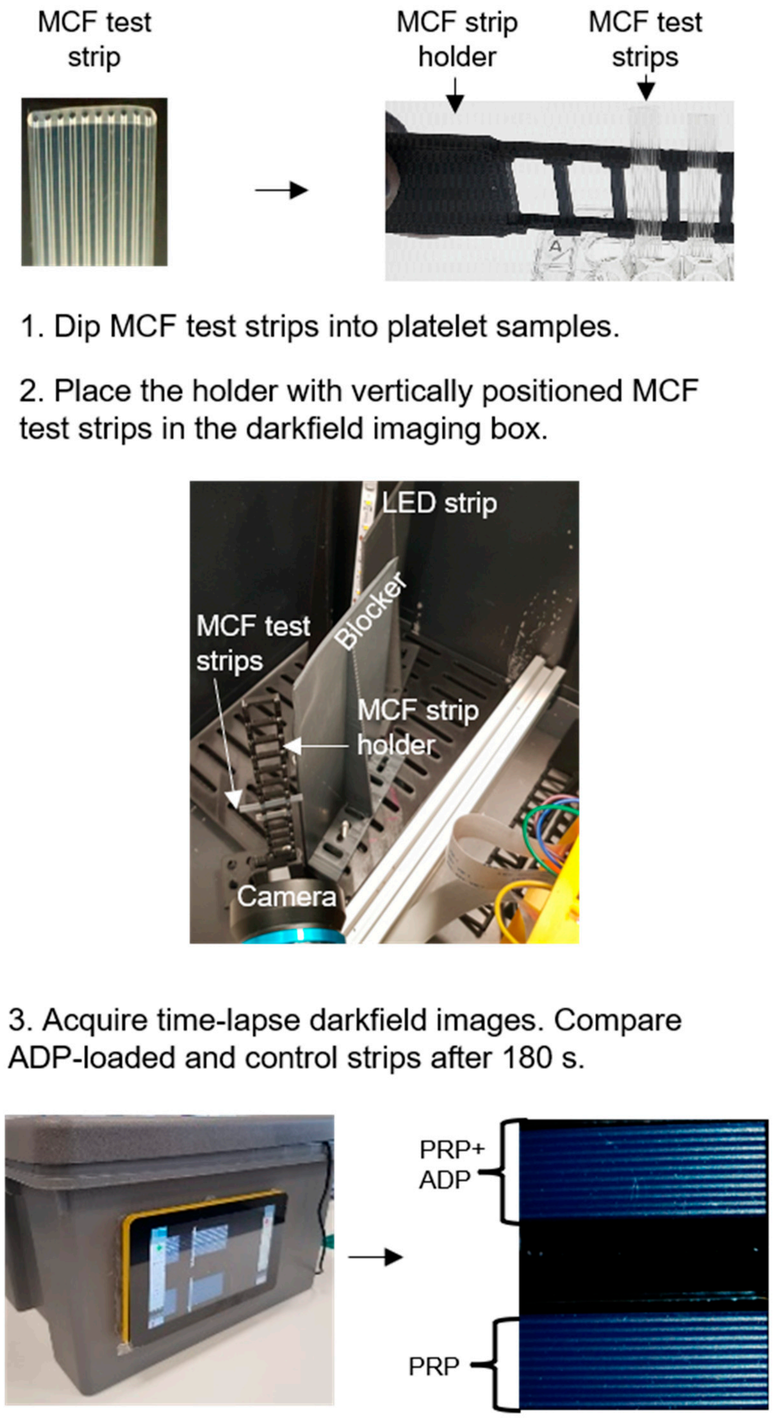

Figure S1. Dark-Pi setup and MCF test strip positioning. MCF test strips were dipped into platelet samples, placed vertically in the holder inside the Dark-Pi imaging box for time-lapse darkfield imaging. Representative darkfield images show the ADP-loaded strip and the control PRP strip after 180 s.

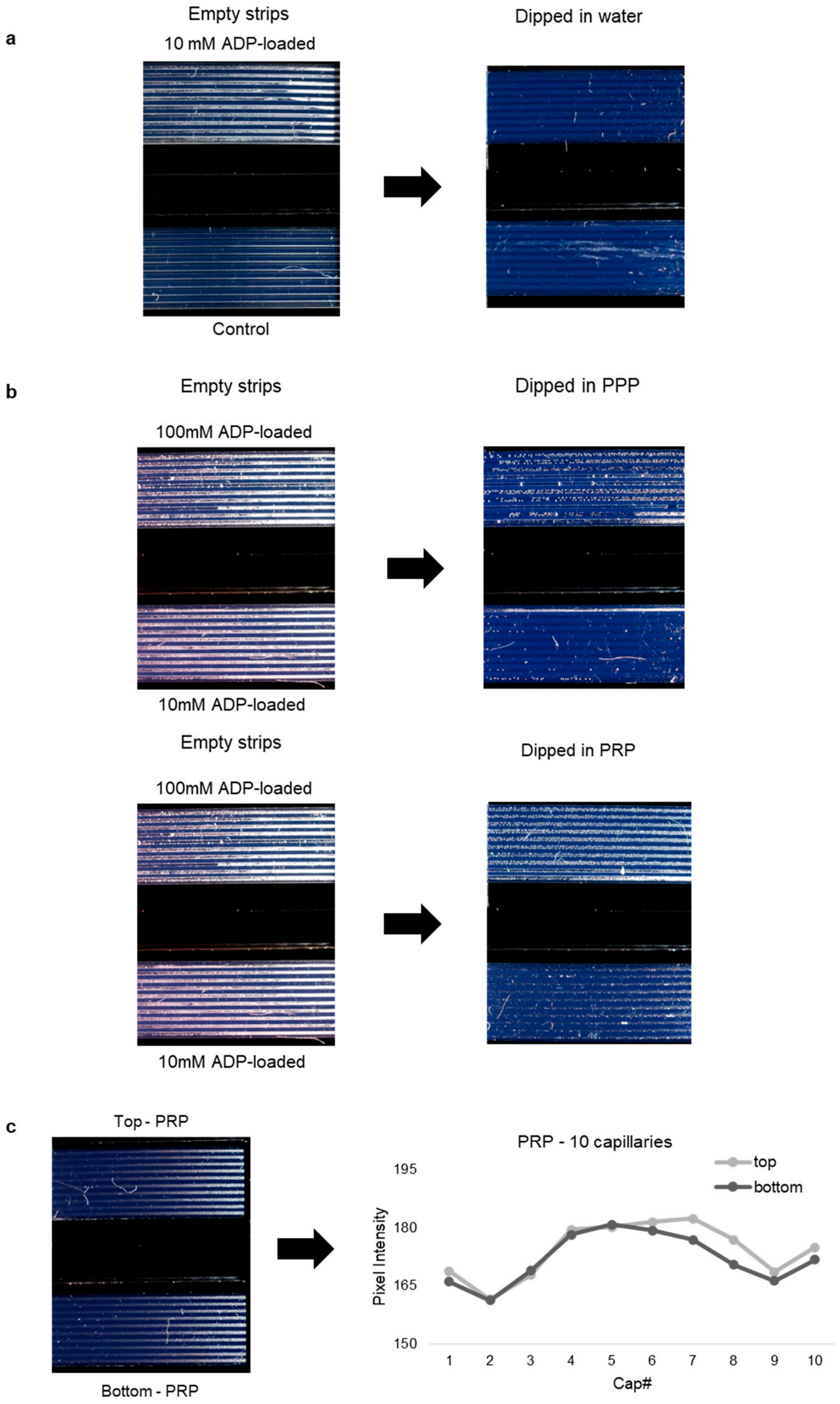

Figure S2. Assessment of crystallization in ADP-loaded and control strips using Darkfield imaging. a) To determine if crystallization occurred in capillaries rather than in platelets, ADP-loaded and control strips were dipped in water after freeze-drying. Darkfield imaging showed no crystal structures in the capillaries. b) We then tested whether higher ADP concentrations would induce crystallization. Strips

with 100 mM ADP displayed crystal structures in both PPP and PRP samples, while those with 10 mM ADP showed no crystals in PPP, indicating that the observed structures in PRP were platelets. c) To optimize the darkfield imaging, results were compared based on the placement of control strips. Minor differences were noted, but overall results were consistent.

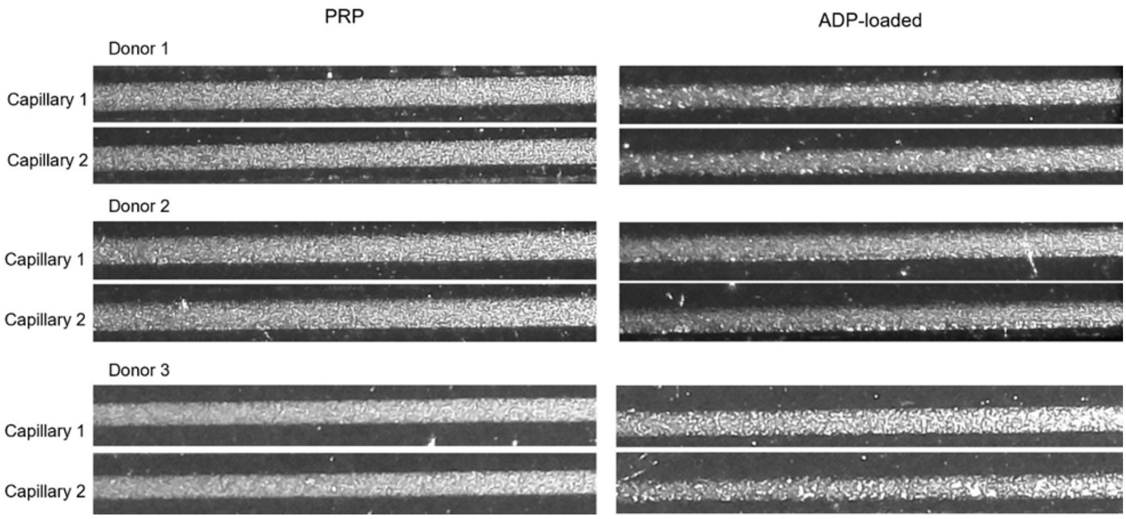

Figure S3. Example images of capillaries with resting vs. aggregated platelets. Samples taken from three different donors were examined after three minutes for two capillaries each selected from ADP-unloaded and loaded test strips dipped in PRP.

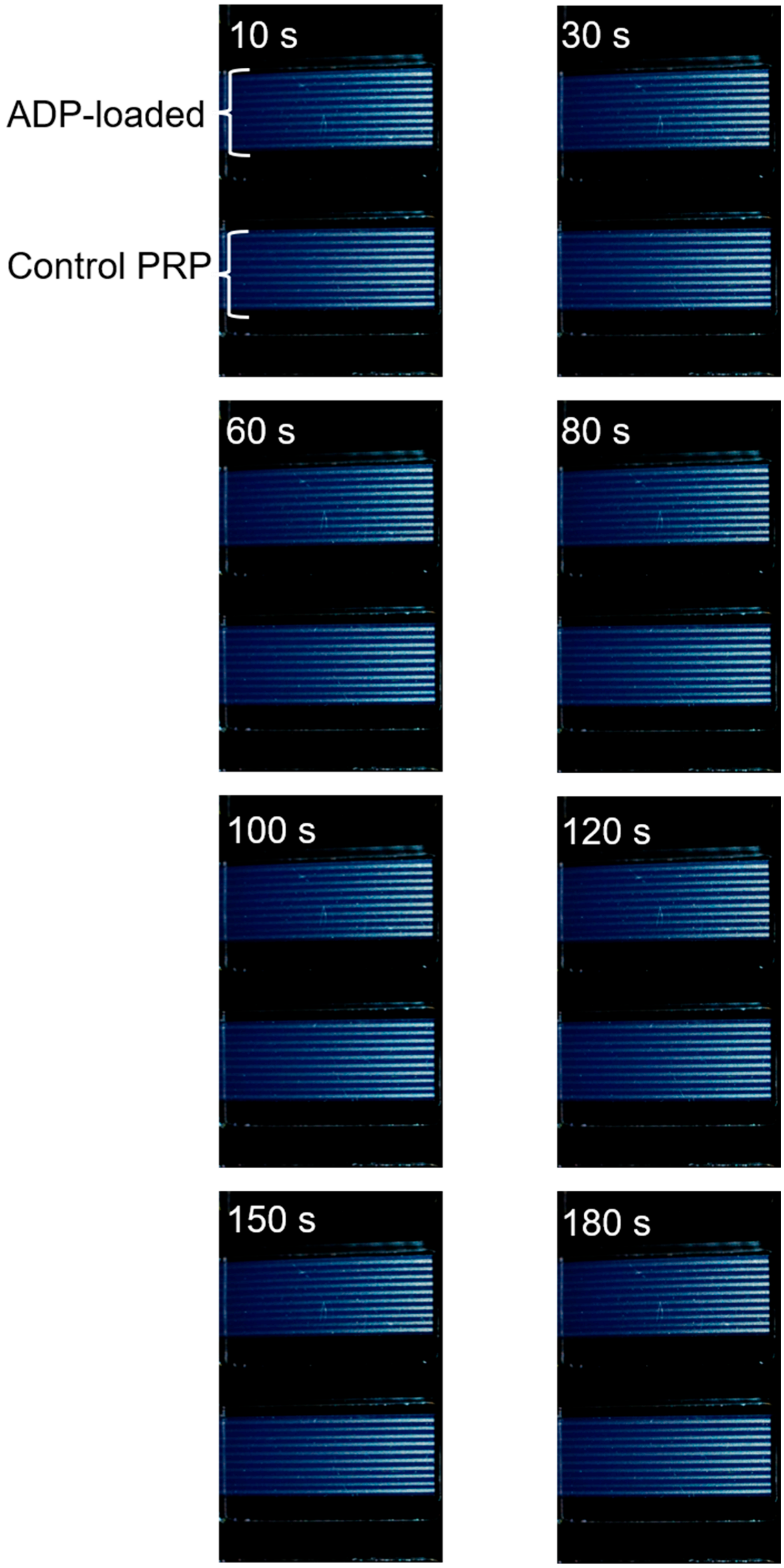

Figure S4. Representative raw time-lapse darkfield images. Eight selected frames from the 3 min acquisition period are shown for control and ADP-loaded microcapillary film test strips. The selected frames illustrate the raw image progression over time.

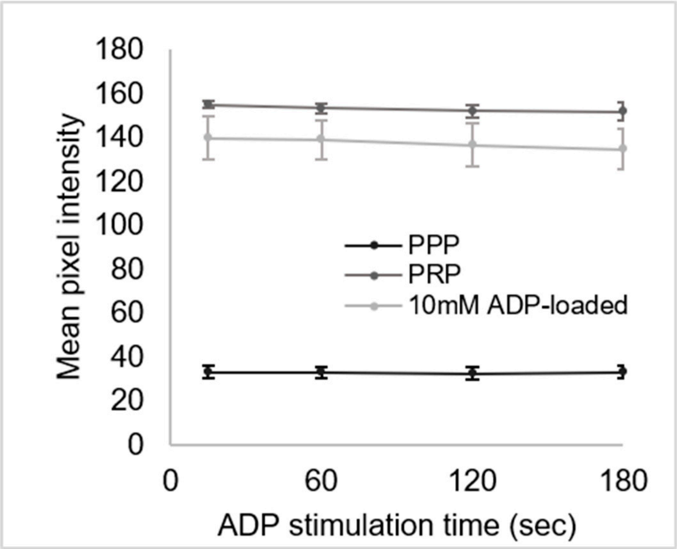

Figure S5. Platelet stimulation within ADP-loaded microcapillary film test strips. The histogram analysis results obtained from the PPP, PRP and PRP in the presence of 10 mM ADP are shown (n = 3 biological donors).

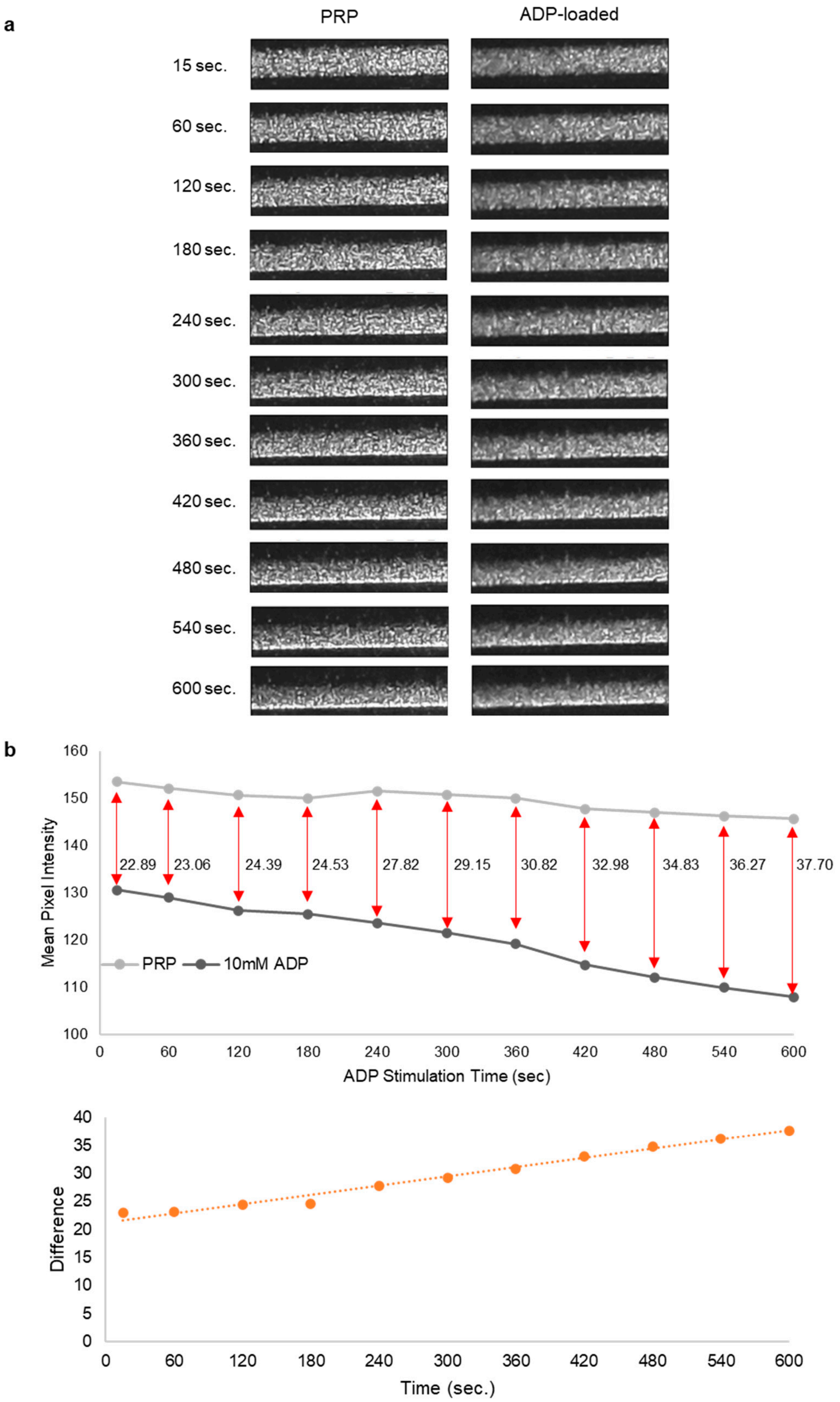

Figure S6. Platelet stimulation in microcapillary film test strips for 10 minutes. a) Images of PRP samples in a selected capillary for one donor acquired every minute for 10 minutes with and without ADP. Thin lines show individual donor traces; thick lines show mean ± SD for PRP control and ADP-loaded MCF strips. b) The increase in the difference between samples over 10 minutes is related to aggregation and sedimentation in the capillaries. It is not the same as saying that platelets only form

aggregates within 10 minutes; aggregates also sediment after a while. Time-lapse images of a selected capillary acquired every minute for 10 minutes suggested that sedimentation started gradually after the third minute. The difference between PRP and ADP also gradually increased after the third minute.

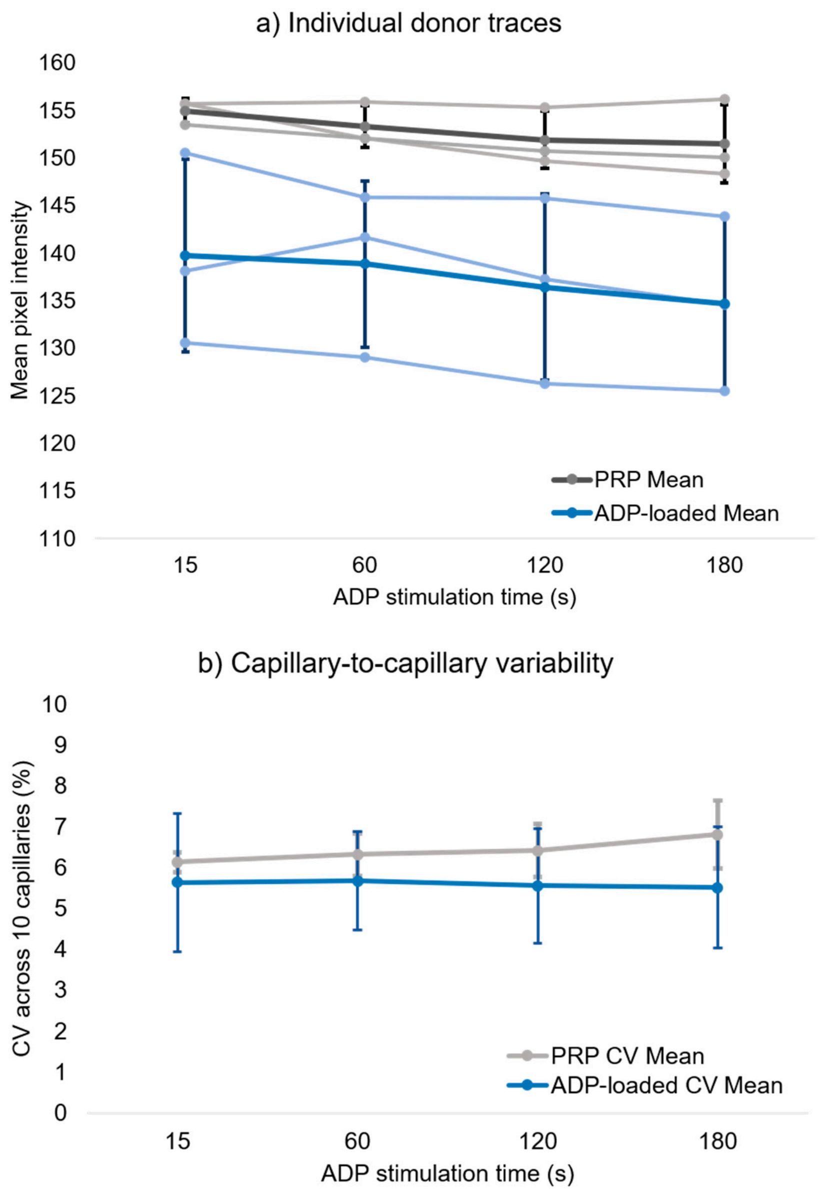

Figure S7. Donor-level and capillary-level Dark-Pi intensity data used for platelet aggregation analysis. a) Individual donor-level mean pixel intensity traces for PRP control and ADP-loaded MCF strips from the three biological donors used in Figure 3B. Thin lines show individual donor traces, and thick lines show the mean. Error bars show the standard deviation across the three biological donors. b) Capillary-to-capillary variability was calculated as coefficient of variation (CV, %) from ten capillaries per condition for each donor. Error bars show the standard deviation across the three biological donors.

Table S1. Capillary-level Dark-Pi mean pixel intensity data from seven analyzed time points.

Donor	Condition	ADP stimulation time (sec)	Cap 1	Cap 2	Cap 3	Cap 4	Cap 5	Cap 6	Cap 7	Cap 8	Cap 9	Cap 10	Mean	SD	CV (%)
1	PRP	15	161,86	136,64	158,09	167,26	159,68	161,41	158,12	152,38	138,24	163,17	155,68	9,83	6,32
1	PRP	30	164,23	135,98	158,21	169,95	159,42	160,16	157,00	150,71	137,16	161,78	155,46	10,54	6,78
1	PRP	60	158,45	132,51	153,37	161,09	158,04	159,04	156,34	147,72	132,53	161,49	152,06	10,48	6,89
1	PRP	90	159,49	133,86	151,66	165,36	154,12	157,41	152,28	148,01	130,65	160,36	151,32	10,65	7,04
1	PRP	120	154,58	132,40	150,25	160,52	154,64	157,72	153,37	144,73	128,53	160,02	149,67	10,59	7,08
1	PRP	150	157,82	131,85	149,47	160,93	151,09	155,88	149,35	143,89	126,50	156,92	148,37	10,76	7,25
1	PRP	180	154,25	131,57	150,62	158,00	155,97	156,89	151,43	141,46	123,81	159,43	148,34	11,52	7,77
1	ADP-loaded	15	162,41	144,54	149,35	156,89	151,30	153,59	151,52	146,96	140,23	148,79	150,56	5,93	3,94
1	ADP-loaded	30	162,71	141,38	147,63	155,71	149,07	154,84	151,25	144,44	140,55	148,60	149,62	6,49	4,34
1	ADP-loaded	60	160,47	139,92	144,89	151,49	147,39	147,55	145,04	141,01	135,42	145,36	145,85	6,48	4,44
1	ADP-loaded	90	160,36	139,74	145,91	151,53	144,35	151,29	145,42	139,95	136,20	148,61	146,34	6,67	4,56
1	ADP-loaded	120	157,31	141,29	144,17	151,06	146,43	148,28	145,50	141,13	134,45	148,11	145,77	5,88	4,03
1	ADP-loaded	150	158,87	138,96	144,51	151,64	143,00	151,71	144,17	138,44	135,31	151,78	145,84	7,06	4,84
1	ADP-loaded	180	155,41	138,53	143,24	145,77	143,47	146,89	143,23	138,52	133,92	149,59	143,86	5,79	4,03
2	PRP	15	161,45	143,26	153,52	161,93	157,13	158,67	155,28	147,82	138,72	156,68	153,45	7,37	4,80
2	PRP	30	160,28	142,52	152,85	160,97	156,44	157,93	154,61	147,19	138,20	155,70	152,67	7,24	4,74
2	PRP	60	161,32	141,87	153,29	159,55	156,34	156,05	152,21	148,32	136,56	155,42	152,09	7,40	4,86
2	PRP	90	159,84	140,94	152,17	158,92	155,48	155,06	151,39	147,29	135,75	159,45	151,63	7,71	5,08
2	PRP	120	160,02	139,82	150,56	159,86	155,59	154,06	151,62	147,00	136,49	152,00	150,70	7,37	4,89
2	PRP	150	158,90	138,93	149,68	158,73	154,41	153,21	150,74	146,21	135,62	156,67	150,31	7,57	5,04
2	PRP	180	157,64	141,95	150,93	158,55	154,52	156,02	150,23	146,21	135,56	148,68	150,03	6,92	4,61
2	ADP-loaded	15	137,32	116,96	137,48	144,09	137,26	135,11	132,59	123,10	112,53	129,13	130,56	9,56	7,32
2	ADP-loaded	30	136,28	116,03	136,39	143,02	136,28	134,19	131,55	122,20	111,64	132,72	130,03	9,54	7,33
2	ADP-loaded	60	136,93	115,23	134,25	141,52	137,60	134,15	130,09	120,55	112,34	127,68	129,03	9,43	7,31
2	ADP-loaded	90	135,84	114,36	133,49	140,38	136,62	133,03	129,18	119,60	111,28	134,22	128,80	9,56	7,42
2	ADP-loaded	120	134,33	112,56	132,20	139,86	133,08	131,60	128,31	116,62	109,57	124,95	126,31	9,62	7,62
2	ADP-loaded	150	134,11	112,42	131,87	139,66	132,84	131,31	128,14	116,46	109,36	124,81	126,10	9,59	7,61
2	ADP-loaded	180	131,73	111,22	130,25	138,85	136,56	128,23	127,13	116,26	109,43	125,38	125,50	9,59	7,64
3	PRP	15	157,85	135,63	153,69	158,43	160,15	161,38	163,51	160,08	141,72	164,89	155,73	9,12	5,85
3	PRP	30	157,23	134,95	152,70	155,71	159,60	160,47	162,97	161,76	141,54	164,53	155,15	9,19	5,92
3	PRP	60	158,59	135,45	154,01	155,78	161,12	161,33	163,80	158,46	143,28	166,94	155,88	9,14	5,87
3	PRP	90	155,38	134,28	153,61	155,43	162,15	160,69	163,38	160,72	141,44	164,50	155,16	9,44	6,09
3	PRP	120	155,80	137,86	154,34	153,69	161,37	164,50	165,61	155,17	141,18	163,67	155,32	8,97	5,78
3	PRP	150	155,04	136,43	154,48	159,82	162,64	159,78	164,73	163,10	144,20	167,30	156,75	9,20	5,87
3	PRP	180	158,39	136,66	154,56	160,87	161,20	163,63	162,73	156,02	139,40	168,11	156,16	9,79	6,27
3	ADP-loaded	15	151,88	121,56	142,87	144,55	146,31	144,07	140,96	135,01	119,18	134,31	138,07	10,10	7,32
3	ADP-loaded	30	152,13	122,80	145,36	147,10	150,47	146,84	144,79	137,86	120,67	136,55	140,46	10,44	7,43
3	ADP-loaded	60	152,24	123,54	145,69	147,28	149,34	146,71	142,21	139,74	122,06	139,44	140,83	9,79	6,95
3	ADP-loaded	90	148,19	121,42	141,69	145,87	146,87	144,18	139,75	134,37	118,68	134,62	137,56	9,85	7,16
3	ADP-loaded	120	151,18	124,20	141,57	143,42	144,92	141,65	138,29	133,75	118,46	134,97	137,24	9,33	6,80
3	ADP-loaded	150	148,80	121,49	139,69	139,57	143,58	139,91	137,28	132,17	116,56	131,89	135,09	9,36	6,93
3	ADP-loaded	180	147,74	119,53	138,28	142,27	141,92	139,65	135,44	129,94	117,03	133,87	134,57	9,40	6,98

Note: Cap = capillary; SD = standard deviation; CV = coefficient of variation. Mean, SD and CV (%) were calculated from ten capillaries for each donor, condition and time point.

Disclaimer/Publisher's Note: The statements, opinions and data contained in all publications are solely those of the individual author(s) and contributor(s) and not of MDPI and/or the editor(s). MDPI and/or the editor(s) disclaim responsibility for any injury to people or property resulting from any ideas, methods, instructions or products referred to in the content.
